# Development and Validation of PCR-Based Ancient DNA Analysis for Italian and Western Balkan Archaeological Collections

**DOI:** 10.2478/bjmg-2026-0008

**Published:** 2026-10-14

**Authors:** M Imran, N Capodicasa, V Barbacovi, PE Maltese, M Bertelli

**Affiliations:** MAGI EUREGIO, 39100 Bolzano, Italy.; MAGI Balkans, Tirana 1019, Albania.; MAGI’S LAB, 38068 Rovereto, Italy.; Tiroler Landesmuseen / Tyrolean State Museum, Archäologische Sammlung / Department of Archaeology.; MAGISNAT OMICS LLC, Atlanta Tech Park, Peachtree Corners,GA 30092, USA.

**Keywords:** ancient DNA, archaeological collections, Western Balkan, mitochondrial DNA, PCR, population genetics

## Abstract

**Introduction:**

The Italian Peninsula and Western Balkans preserve rich archaeological records that extend from pre-Roman times through the Greek colonial, Roman, and Byzantine periods. Genetic investigation of these collections often requires access to specialized high-throughput technologies which are not commonly available or cost-effective method to handle the problem of broken skeletal remains.

**Methods:**

We developed and validated an ancient DNA protocol for Italian and Albanian archeological skeletal samples spanning approximately 2500 years. The protocol included artificial fragmentation of modern DNA to simulate ancient molecular profiles and validation using archaeological remains from Sicily, Rome and Apollonia. The workflow integrated EDTA demineralization, silica-based purification, PCR targeting short amplicons (<150 bp) to enable sex determination, species authentication, uniparental haplogroup analysis, and pigmentation trait prediction. Strict anti-contamination and authentication criteria were enforced.

**Results:**

Amplifiable DNA was recovered from all tested skeletal samples. Mitochondrial DNA haplogroups were assigned in 82%, while Y-chromosome markers were found in 76% of males, while sex was determined in 85% of the samples. Pigmentation SNPs yielded complete profiles in 78%. Amplicons <110 bp showed >90% amplification success. All results were reproducible and met authentication criteria, including phylogenetic plausibility and inverse fragment length-yield correlation.

**Conclusion:**

This protocol enables ancient DNA analysis from degraded skeletal remains of Mediterranean archaeological contexts. The simulated ancient DNA presents a key innovation, allowing refinement of procedures before application to irreplaceable material and provides a practical approach for laboratories with limited access to High-Throughput sequencing. The research is a practical framework for studying mobility, ancestry and demography among ancient populations from the Mediterranean and Western Balkan regions.

## INTRODUCTION

The Italian Peninsula and the Western Balkans are among the archaeologically rich regions of the Mediterranean, with skeletal collections spanning critical periods of European demographic and cultural history. The Italian archaeological heritage spans pre-Roman indigenous populations who included Etruscans, Daunians, other Italic groups, and Greek colonization of Magna Graecia which began in the 8th century BCE through Tarentum, Sybaris, Croton, and Phoenician coastal establishments and Roman territorial expansion which continued from the Republican through Imperial periods and Byzantine administration [ 1, 2, 3, 4]. The Western Balkans, particularly present-day Albania, witnessed parallel historical trajectories which included Iron Age Illyrian populations and Greek colonial foundations along the Adriatic and Ionian coasts which established Apollonia around 600 BCE through Corinth and Epidamnos and Roman conquest and integration and the subsequent establishment of Byzantine control [5,6].

Recent ancient DNA studies have begun to show the genetic effects which followed these historical events. The genomic analysis of Etruscan remains showed no signs of recent Anatolian ancestry but it did show steppe-related ancestry which dated back to the Iron Age period and the Roman Imperial period brought about complete population changes through 50 percent of eastern Mediterranean ancestry [2]. The study of Bronze Age and Iron Age central Italy showed that steppe-related ancestry made its first appearance in 1600 BCE and then became more common over time alongside changes in burial customs and kinship systems [3]. The research on southern Italian Iron Age people who lived at that time studied Daunian people from Apulia which discovered that their genetic makeup differed from both modern-day Roman people and the broader Mediterranean genetic system [7,8]. Greek settlement patterns about Sicily and southern Italy discovered through dental and ancient DNA research that different demographic effects occurred which showed some studies found few Greek immigrants who settled among local residents while other studies established definite genetic evidence of Euboean settlement in eastern Sicily [9,10]. The archaeological research and genetic studies of Greek-Illyrian interactions between Greece and Albania have conducted biodistance research through dental and cranial measurements at Apollonia to determine how population groups interacted with each other [5]. Recent genetic research on the populations of southern Italy, Sicily, Greece, and Albania has shown that all these groups share a common Mediterranean genetic heritage which includes southern Italian people showing closer genetic ties to Greek islands than to the mainland of Greece while their ancestry shows elements from post-Neolithic Levantine and Caucasus populations who migrated during the Bronze Age maritime time period [6].

The existing scientific research about Italian and Balkan archaeological sites has mostly concentrated on genetic studies which focus either on remains that date back less than 2000 years or on advanced sequencing technologies which require extensive specialized equipment and funding. The scientific community needs to develop better protocols which researchers can access to conduct genetic testing on skeletal remains that date from pre-Roman times until the Byzantine era which lasted 2500 years.

The study of ancient DNA from skeletal remains presents scientists with special difficulties during their research. The process of DNA degradation which occurs after death, progresses through enzymatic breakdown and hydrolytic cleavage and depurination and cytosine deamination, leads to fragmentation that results in DNA segments which range between 100 and 200 base pairs [ 11, 12, 13, 14]. The process of DNA decay exhibits complicated patterns which include cytosine deamination that follows thermal age models while DNA fragmentation reaches its maximum speed and then decreases, thus demonstrating that burial conditions create systems which effectively maintain DNA integrity[13,14]. The ability to preserve DNA depends on environmental conditions which include temperature and humidity together with soil pH and geochemical characteristics and microbial activity, while DNA remains intact under conditions of dryness and low temperature and slightly alkaline environments and total lack of microorganisms [11,15,16]. DNA preservation in Mediterranean climates presents particular challenges due to elevated temperatures, yet studies of Italian archaeological sites which exist from the 9th–12th centuries CE and earlier periods have shown that scientists can successfully extract amplifiable DNA when they choose proper extraction techniques and scientific methods [12,17]. The majority of bone DNA resides within osteocytes which are embedded in calcified matrix because the DNA molecules attach themselves to hydroxyapatite crystals, which requires demineralization to enable proper extraction [12,16,18]. Researchers have found that the petrous portion of the temporal bone and tooth cementum function as the best sampling sites because these locations provide researchers with 16–40% endogenous DNA content which exceeds the 2–3% found in other skeletal elements, yet the results from different samples show high variability and researchers can assess preservation quality through visual inspection [18, 19, 20].

The objective of this study was to develop, optimize, and validate a comprehensive ancient DNA analysis protocol specifically applicable to archaeological skeletal remains from Italy and the Western Balkans. Through extensive bibliographic survey of published methodologies, consultation with laboratories specializing in archaeogenetics and forensic DNA analysis, and systematic experimental optimization, we established standardized methods suitable for museum and excavation collections spanning approximately 2500 years of Mediterranean and Balkan history. The protocol addresses species authentication, biological sex determination, and uniparental genetic markers (mitochondrial and Y-chromosomal DNA) and phenotypic trait prediction, providing a practical framework for laboratories engaged in archaeological genetic research with limited access to high-throughput sequencing infrastructure.

## MATERIALS AND METHODS

### Study design & sampling

Protocol optimization was performed using modern DNA reference samples (Coriell Laboratory) in a dedicated ancient DNA facility with physically separated pre-PCR and post-PCR areas. To approximate ancient DNA molecular characteristics, modern reference DNA was subjected to controlled degradation designed to reduce fragment length to the 200–250 base pair range typical of well-preserved archaeological samples from the last 2000–2500 years. Selected experiments combined fragmented reference DNA with protein-rich matrix prior to extraction to simulate the physical association of DNA with bone matrix components including collagenous proteins and hydroxyapatite [12,16]. Validation employed anonymous ancient skeletal samples from Italian archaeological sites including Himera (Sicily), Baucina (Sicily), and Portico di Ottavia (Rome). In the case of the Western Balkan area, the archaeologically collected bone samples used in the present protocol were from Apollonia, Fier region, Albania. Sampling was performed using a minimal-destruction approach and only material approved for molecular analysis was processed. No living participants were involved. Sample identifiers were coded and linked to archaeological metadata only through institutional records. All analyses conducted were performed under the appropriate national laws and institution-specific guidelines for human remains and ancient DNA contamination studies. The genetics lab did not participate in any way in the excavations nor in the recovery of the bones. Bone fragments were made available for the laboratory work by our collaborating archaeologists. The protocol was intended for use on Balkan archaeological material, specifically Albanian sites like Apollonia, where taphonomic circumstances, burial traditions, and temporal antiquity conditions were identical to those of the Italian control material [5]. The protocol workflow design and analysis setup were presented in**Figure 1** and **Table 1**.

**Figure 1.**
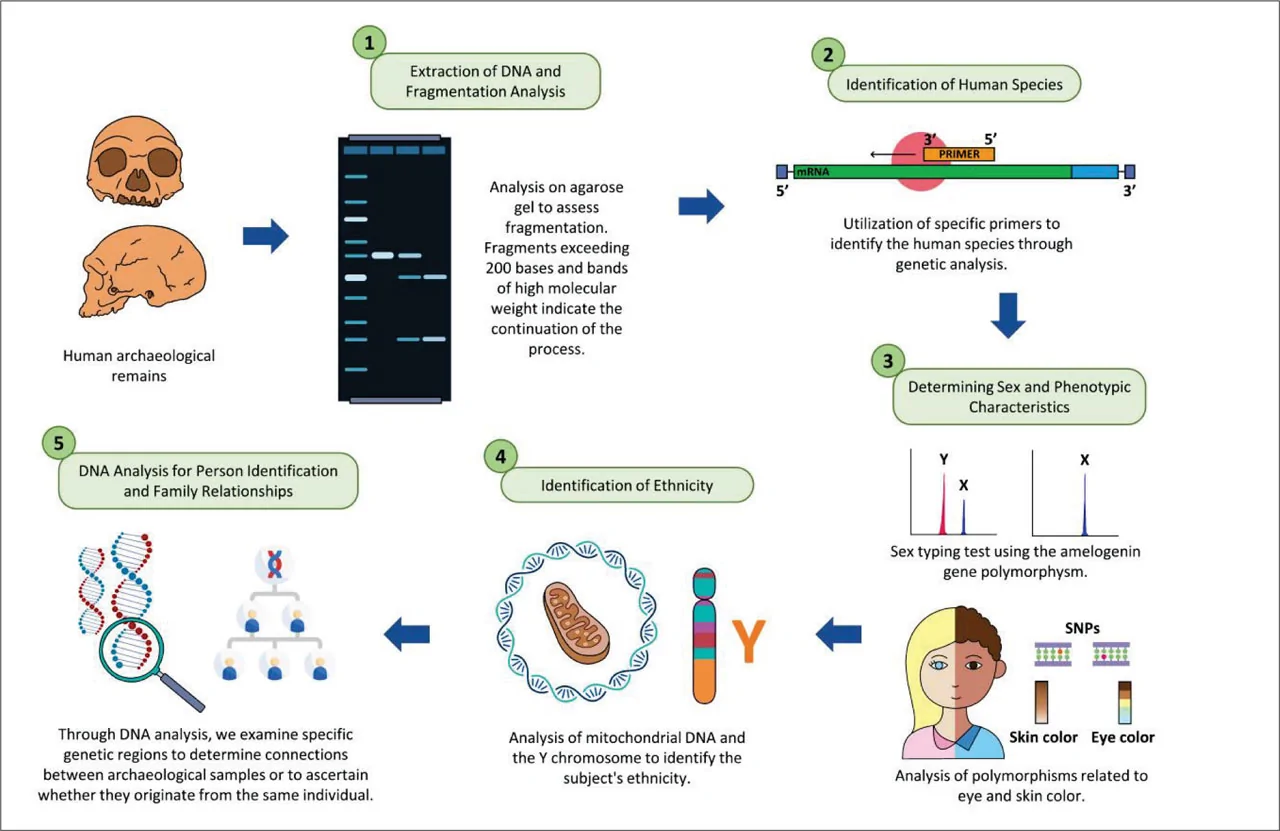
Overview of the protocol workflow.

**Table 1.** Setup of the protocol analysis summarized in various steps.

**Analysis Step**	**Target**	**Primer Name**	**Primer Sequence (5′-3′)**	**Information Provided**	**References**
**DNA Extraction Sample Preparation**					
Bone surface cleaning	N/A	N/A	N/A	UV irradiation; physical removal of outer layers to reduce contamination	12,27, 28, 29
Bone powder preparation	N/A	N/A	N/A	Grinding of cleaned bone samples	12,27,30
Demineralization	N/A	N/A	EDTA 0.5M pH 8.0	Decalcification of bone matrix; releases DNA from hydroxyapatite	12,30,31
Protein digestion	N/A	N/A	Proteinase K (20 mg/mL) + 10% SDS	Cell lysis; protein degradation	12,27,31
DNA purification	N/A	N/A	QIAquick PCR Purification Kit (silica-based)	Removes PCR inhibitors; concentrates DNA	12,27,30
**Data Integration/Interpretation**					
PCR product purification	N/A	N/A	Gene Matrix PCR/DNA Clean-Up Kit	Removes primers and dNTPs before sequencing	21
Sequencing	N/A	N/A	Sanger sequencing (≥144 bp); Cloning + sequencing (144 bp)	Determines nucleotide sequence for genotyping	21,32
Authentication	N/A	N/A	N/A	Reproducibility testing; inverse fragment size/success relationship; phylogenetic coherence; negative controls	33,34
Haplogroup assignment	mtDNA	N/A	PhyloTree Build 17	Assigns maternal haplogroups based on HVR sequences	35
Haplogroup assignment	Y-chromosome	N/A	YFull tree	Assigns paternal haplogroups based on Y-SNPs	1,36,37
Phenotype prediction	Pigmentation SNPs	N/A	HIrisPlex/HIrisPlex-S systems	Predicts eye, hair, and skin color from genotype data	30
Population analysis	Combined data	N/A	N/A	Integrates all genetic data to reconstruct individual profiles and population history across 2500 years of Italian demographic changes	6,36,38

### Ancient sample preparation and DNA extraction

All ancient samples were processed following strict contamination prevention protocols including UV irradiation of work surfaces, dedicated equipment, extraction and PCR negative controls, and physical separation of pre- and post-amplification procedures. Bone surfaces were first cleaned by physical removal of around 1–2 mm from the outer cortex. The fragments were subjected to washing in 0.5% sodium hypochlorite (NaOCl) for 15 min, three washes with ultrapure water, UV irradiation for 30 min each side at 254 nm, and grinding to obtain fine bone powder. Around 200–500 mg of bone powder was demineralized in 0.5 M EDTA, pH 8.0, for 48–72 h at room temperature with continuous rotation. Demineralized bone material was digested overnight at 56°C using proteinase K (20 mg/mL) and 10% SDS, followed by silica-based purification using QIAquick PCR Purification Kit (Qiagen) [12,27, 28, 29, 30, 31]. Blanks were processed along with the archaeological samples, while negative controls without template DNA were included in all PCR reactions.

### Targeted marker panel selection

Extensive literature review was done for primer selection and it was also guided by methodological exchanges with archaeogenetics and forensic DNA analysis laboratories. Emphasis was on the markers validated for degraded DNA, short amplicon length (<150 bp), and high specificity under low-template conditions. Published protocols were encompassed by comprehensive bibliographic survey for ancient DNA extraction, PCR optimization for degraded samples, authentication criteria, and selection of genetic marker for archaeological applications spanning the past two decades of methodological development in the field.

Amelogenin gene length polymorphism was utilized for sex determination with primers Amelogenin-F, Amelogenin-R, and SRY gene detection with amplicons <60 bp [21]. Mitochondrial 16S rRNA gene (rDNA) of vertebrates was targeted by specific primers for species authentication. These primers were specifically designed for the amplification of eukaryotic mitochondrial DNA and also show multiple mismatches with prokaryotic 16S rRNA genes which stop bacterial amplification, while they enable accurate identification of human and non-human vertebrate remains.

SNPs were used for the prediction of phenotypic traits related to pigmentation through the application of optimized PCR which generated amplicons that ranged from 96 to 149 bp [12,18,22,23]. The study included target genes and primers, which were presented in the **Table S1**. Mitochondrial DNA hypervariable regions HVR-I and HVR-II were amplified using primers optimized for degraded DNA with amplicons <120 bp[24, 25, 26]. Y-chromosome analysis used SNP markers for major Mediterranean haplogroups which had amplicons that measured less than 100 base pairs[18,24].

### Polymerase chain reaction and gel electrophoresis

Amplifications were conducted in a total reaction volume of 25 µL comprising 1× AmpliTaq Gold buffer, 1.5 µL of 25 mM MgCl_2_ (final concentration 1.5 mM), 0.2 mM of each dNTP, 0.3–0.8 µL of each 10 µM primer, 5% DMSO, 0.8 mg/mL BSA, 1.25 U AmpliTaq Gold DNA polymerase, and 2–5 µL of template DNA. Thermal cycling involved an initial activation at 95°C for 10 min followed by 33–38 cycles of denaturation at 94°C for 30 s, annealing at 60–65°C for 30 s, extension at 72°C for 30 s, and a final extension at 72°C for 7 min. PCR products were analyzed by gel electrophoresis on 2–3% agarose gels at 100 V for 45–60 min using 50-bp DNA ladder. PCR products were purified using Gene Matrix PCR/DNA Clean-Up Kit. Sanger sequencing was applied to fragments that measured 144 base pairs or more while fragments shorter than that needed cloning to achieve consensus reconstruction [40] PhyloTree Build 17 was used to classify sequences which assigned haplogroups to various European and Mediterranean populations [12,40, 41, 42]. Authentication proceeded according to established criteria which required reproducibility through independent extractions and proved inverse DNA quantity relationship with fragment length and showed phylogenetic coherence with established populations and was verified through negative control testing [43].

### Statistical analysis

Quantitative laboratory data was organized in a structured excel workbook before analysis, where variables include analytical module, target gene or region, marker ID, amplicon size, PCR or sequencing outcomes, gel-lane classification, and negative-control status. Summary outcomes of performance were analyzed separately from sample-level experimental data to avoid mixing aggregate percentages with raw sample-marker observations. All analyses were performed in R, where data were imported using the packages and visualized in the form of plots. Protocol performance was summarized as percentages for species authentication, overall amplification, sex determination, mitochondrial/Y-chromosome haplogroup assignment, pigmentation profiling, reproducibility, and negative-control performance. When raw numerators and denominators were available, Wilson 95% confidence intervals (CIs) were calculated for binomial proportions. When only percentages were available, results were treated descriptively and no inferential CIs were reported.

The design of the amplicon was evaluated by plotting expected product sizes for each marker and by comparing amplification success between short amplicons (<110 bp) and longer amplicons (>130 bp). Meanwhile, the results of the gel electrophoresis were summarized using lane-level visual scoring of sample and negative-control bands. Lanes were classified as band present, no band, or unclear. Because gel-band scoring was based on visual inspection, these results were considered preliminary pending densitometric confirmation. Sequencing-optimization information was retained as supplementary methodological evidence.

## RESULTS

Protocol optimization using modern DNA samples established baseline performance parameters for all genetic markers. Optimization experiments using artificially degraded reference DNA yielded amplification profiles comparable to those obtained from well-preserved ancient skeletal samples, with PCR success strongly dependent on fragment length. Targets within the 200–250 bp range showed higher reproducibility and sensitivity than longer amplicons, aligning with expectations for archaeological material dating to approximately 2000 years before present and supporting the suitability of the optimized protocol for moderately degraded ancient DNA.

### Protocol performance and marker amplification

The targeted protocol showed high apparent performance across the main analytical modules, as shown in **Figure 2** or **Table S2**. Species authentication, overall amplification, and negative-control performance were evaluated with reproducibility. Sex determination was successful in 85% of tested samples, mitochondrial haplogroup assignment in 82%, pigmentation profiling in 78%, and Y-chromosome haplogroup assignment in 76% of male samples. These values are presented descriptively because raw numerators and denominators remain to be verified before final CIs can be calculated.

**Figure 2.**
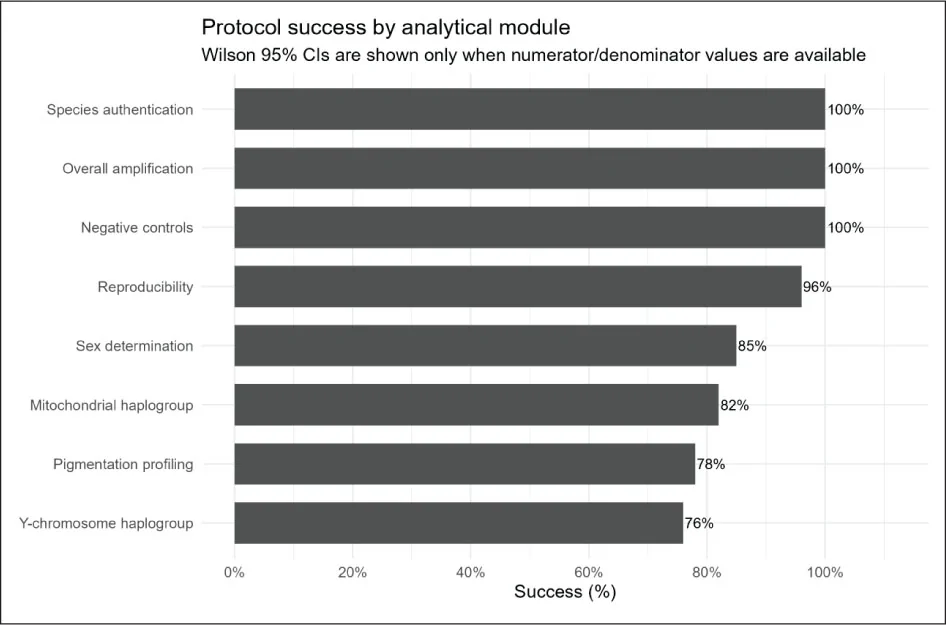
Amplification success and analytical output rates presented by amplicon module.

### Amplicon-size strategy

Amplicon-size analysis supported the short-fragment design of the protocol (**Figure 3**, **Table S3** or **S4**). Amplification success was higher for amplicons <110 bp than for amplicons >130 bp (92% vs 68%), supporting the use of short PCR targets for degraded ancient DNA (**Figure 3A**). The marker panel ranges from approximately 60 bp to 149 bp, with sex-determination markers at approximately 60 bp, several Y-chromosome and 16S targets around 100 bp, pigmentation markers such as *SLC45A2, MC1RB, HERC2, MC1RA, OCA2B*, and *SLC24A5* within the short-to-intermediate range and longer targets including *OCA2A, OCA2D* and *DCT*, as shown in **Figure 3B**.

**Figure 3.**
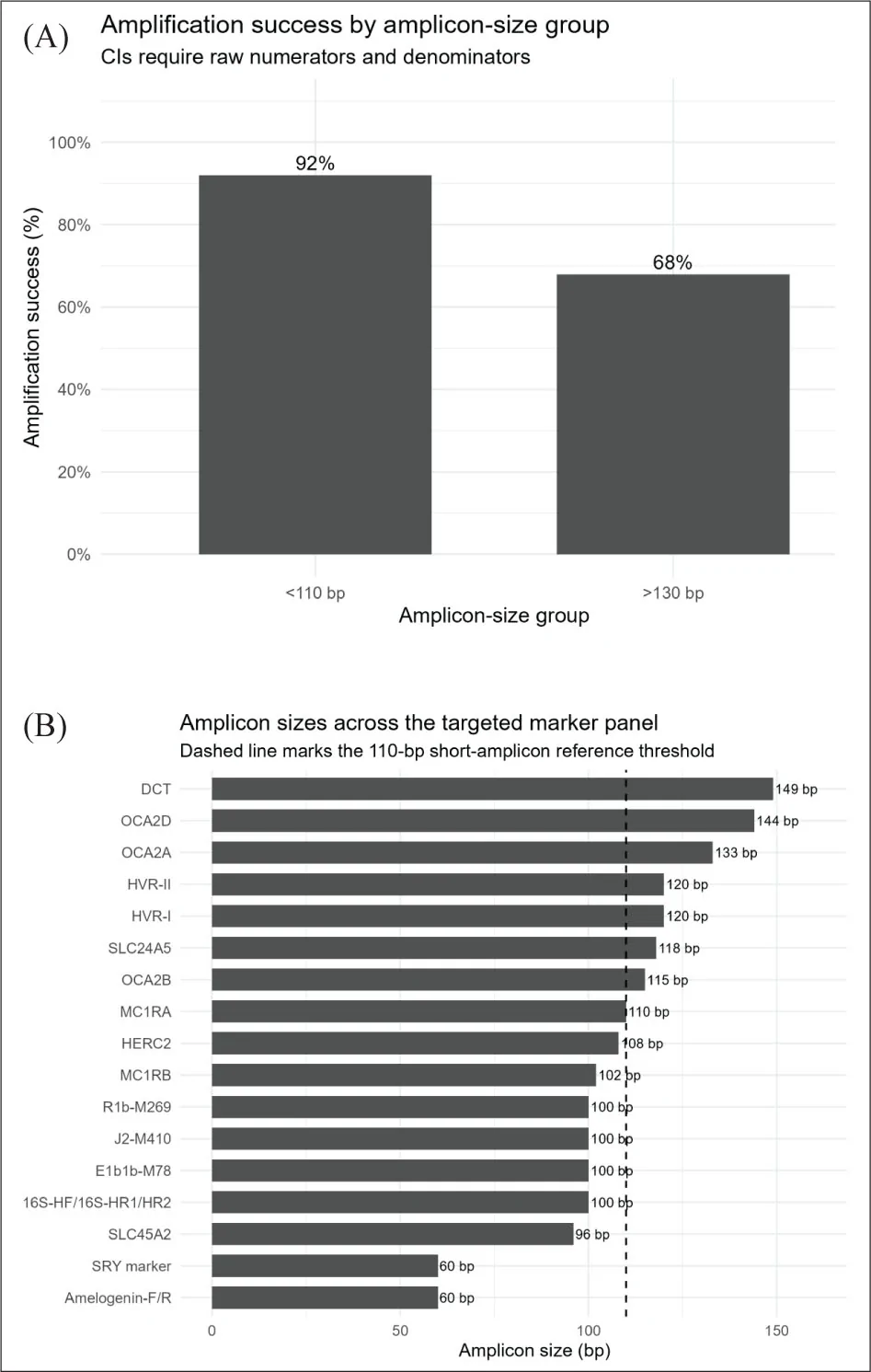
Marker or amplicon size panels representing (A) amplicon success percentage by amplicon-size groups and (B) amplicon sizes across the targeted marker panels.

### Gel-based validation and controls

Lane-based gel scoring showed visible amplification bands for multiple target markers including mitochondrial DNA and pigmentation-associated loci. Representative agarose gel electrophoresis confirmed successful amplification of selected pigmentation and mitochondrial targets, with negative controls showing variable performance across marker panels and experimental runs, as shown in **Figure 4**. No amplification bands corresponding to target size were observed for negative control lanes. Low molecular weight bands that might have been observed were attributed to nonspecific products or primer dimers and excluded from the analysis. Original annotated gels are presented in the **Figure S1**. Direct sequence interpretability analysis demonstrated marker-specific differences **(Figure S2).** Sequences of *DCT* and *OCA2D* and a Mitochondrial target of 1200bp (MT) were considered evaluable. The following markers were listed as not evaluable/unclear: *MC1RA, MC1RB, HERC2, OCA2B, OCA2C, SLC24A5*, and *SLC45A2.* Repeat-sequence combined analysis demonstrated doubtful evaluation result. *OCA2A* was not listed as any elution or PCR product.

**Figure 4.**
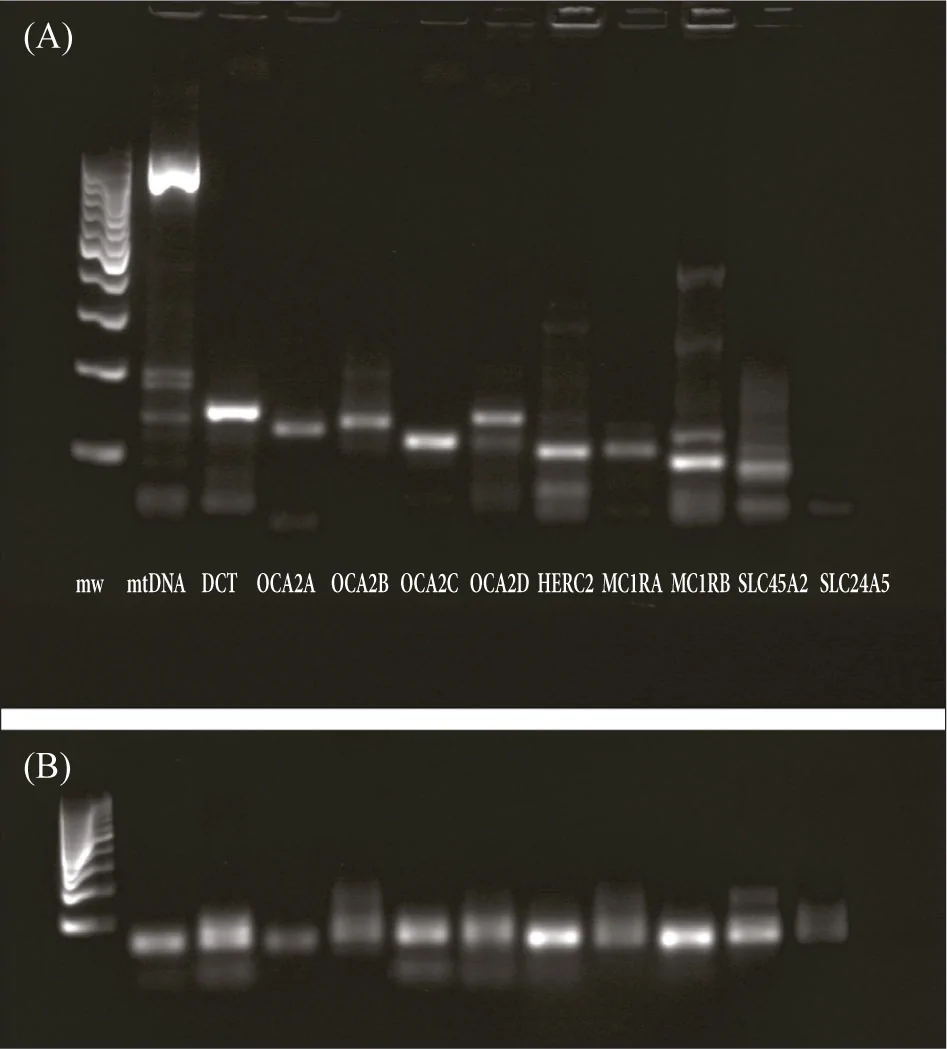
Representative PCR gel electrophoresis showing amplification of (A) short pigmentation /marker targets and (B) Corresponding no-template negative-control reactions assessed for non-specific amplification or contamination.

The optimized extraction protocol successfully recovered amplifiable DNA from ancient skeletal samples across all time periods tested (up to 2500 years before present). Sex determination using amelogenin and *SRY* markers yielded complete profiles from 85% of ancient samples with input DNA as low as 50 pg. Species authentication using 16S rRNA primers successfully distinguished human from animal remains in all tested samples. Phenotypic SNP analysis demonstrated successful amplification of all target loci from ancient samples, with complete profiles obtained from 78% of samples. Amplification success correlated inversely with fragment size, with fragments <110 bp showing 92% success rate compared to 68% for fragments >130 bp [26].

Mitochondrial *HVR-I* and *HVR-II* sequencing successfully assigned haplogroups to 82% of ancient samples tested. Y-chromosome haplogroup determination was successful in 76% of male samples. Negative controls remained negative throughout all experiments. Reproducibility testing showed 96% concordance across independent extractions and amplifications. Authentication criteria were met for all samples, including appropriate molecular behavior (inverse relationship between amplification success and fragment size), phylogenetic coherence with known Mediterranean populations, and reproducibility [43].

## DISCUSSION

This study successfully developed and validated a comprehensive ancient DNA analysis protocol specifically designed for Italian and Western Balkan archaeological collections spanning 2500 years. The protocol addresses the major challenges of ancient DNA analysis including degradation, low copy number, and contamination through optimized extraction methods, short amplicon design, and strict authentication procedures [39,43,44]. A critical innovation of this protocol lies in the use of artificially degraded modern reference DNA to simulate ancient DNA characteristics during optimization. Controlled fragmentation of Coriell reference samples to the 200–250 bp range enabled systematic testing of extraction efficiency, primer performance, and PCR conditions under controlled conditions that closely approximate the molecular properties of well-preserved archaeological samples from Mediterranean burial contexts [39,44]. This approach allowed identification of optimal parameters before applying the protocol to irreplaceable ancient material, reducing the risk of sample loss and improving reproducibility. The combination of fragmented reference DNA with protein-rich matrix further simulated the physical association of DNA with bone components, providing a realistic model for method development that accounts for the organo-mineral interactions characteristic of archaeological bone[12,16].

The silica-based extraction method with EDTA demineralization proved highly effective for recovering amplifiable DNA from highly fragmented ancient skeletal remains. Recent comparative studies have demonstrated that silica-based methods, particularly those employing full demineralization, show superior performance for degraded samples compared to organic extraction methods, with success rates varying by skeletal element and preservation context [12,25,26,43,44]. This size-dependent recovery pattern represents a fundamental authentication criterion for ancient DNA, distinguishing endogenous ancient molecules from modern contaminants which typically exhibit uniform amplification success across fragment sizes and confirming the suitability of the extraction protocol for highly degraded material [43,45]. The protocol was designed to reflect the logical sequence of genetic questions typically addressed in archaeological investigations. Species authentication represents the essential first step, excluding non-human remains and preventing wasteful analysis of faunal material that may be morphologically ambiguous or fragmentary [39]. Following confirmation of human origin, DNA extraction and authentication establish the presence of endogenous ancient DNA through characteristic molecular behavior patterns. Biological sex determination provides fundamental individual-level information essential for demographic reconstruction and kinship analysis [1,17]. Mitochondrial and Y-chromosomal markers enable inference of maternal and paternal ancestry, facilitating investigation of sex-biased migration patterns, marital residence practices, and population continuity across historical transitions [2,3,12]. Pigmentation-associated SNPs allow prediction of externally visible traits, facilitating comparison with archaeological, anthropological, and historical data regarding population appearance and phenotypic diversity [4,5]. Based on the extensive bibliographic survey conducted for this study and the empirical validation results, the optimized protocol appears suitable for approximately 80% of skeletal remains from the target chronological period (2500 years before present to recent historical periods) in Italy and Albania. The estimation depends on academic research studies which examine DNA preservation in Mediterranean archaeological collections where researchers proved that preservation depends on local storage conditions yet most skeletal remains from ancient burial sites in temperate regions can be tested for DNA through specific extraction techniques and short DNA fragment testing methods [6,12,17,21]. The remaining 20% of samples exhibiting poor DNA preservation typically reflect exposure to extreme taphonomic conditions including high temperature fluctuations, acidic soils, prolonged water exposure, or extensive microbial colonization [21,41,42]. The success rates observed in this study (85% for sex determination, 82% for mitochondrial haplogroup assignment, 76% for Y-chromosome analysis, 78% for phenotypic SNP profiling) align closely with published success rates for PCR-based ancient DNA analysis of skeletal remains from comparable chronological periods and geographic contexts[12,17,25]. The results show that the protocol delivers performance results which match established methods while providing laboratories without access to high-throughput sequencing equipment a single standardized framework for multiple analytical target testing.

Researchers can conduct genetic research studies that require more than 2000 years of historical data because they can analyze human remains which date back to 2500 years ago. The genetic research for Italian collections now permits analysis of Etruscans Daunians and other Italic groups which existed before Roman times [18,24,39,44]. This research enables scientists to compare genetic information from these ancient populations with data from Roman and post-Roman times to study how people inter-bred through time[18,39,44]. The protocol enables genetic analysis of Iron Age Illyrian populations and ancient Greek settlements at Apollonia and Epidamnos. The research investigates demographic shifts and Roman-Illyrian interactions which occurred from prehistoric times until the historical period [26,40].

The standardized protocol developed here can be applied systematically to museum and archaeological collections throughout Italy and the Western Balkans. The protocol enables researchers to study skeletal remains from historical mausoleums Christian burial sites Greek necropoleis Roman cemeteries and ongoing archaeological excavations. The protocol can be used in different research settings because Mediterranean and Balkan archaeological sites share similar taphonomic conditions and burial practices and have predictable skeletal preservation rates which match their burial environments and time depths [21,23,42].

A key added value of this work lies in the integration of extensive bibliographic survey, expert laboratory consultation, and experimental validation into a single standardized protocol. By consolidating all major analytical steps and providing empirically tested primer sequences within one methodological framework, this study reduces the need for fragmented, study-specific optimization that characterizes much of the published ancient DNA literature. The resulting protocol offers a practical and reproducible reference for laboratories engaged in archaeological and forensic genetic analyses, particularly for well-preserved to moderately degraded remains dating to the last two to three millennia, and supports interdisciplinary interpretation in conjunction with historical, archaeological, and anthropological evidence.

## CONCLUSION

The current study presents an entire ancient DNA analysis methodology, which was validated by applying it on skeletal samples obtained from Italian and Western Balkan collections dated up to 2500 years old, representing various Mediterranean and Balkan historical periods. Through systematic optimization of ancient DNA protocols on artificial degradation of modern DNA samples, thorough bibliographic reviews on current methodologies, communication with specialized laboratories, and the practical implementation of ancient DNA protocols on samples collected from the Italian archaeological sites, we developed a standard protocol which includes methods for the authentication of species and the identification of biological sex, determination of mitochondrial and Y-chromosomal haplogroups and phenotypes.

We solved the basic problems associated with ancient DNA analysis with the silica-based method of extraction and EDTA demineralization, application of a short (<150 bp) amplicon, optimal PCR conditions for low-template and degraded DNA, and rigorous authentication procedures. We proved that our protocol is highly efficient, reaching success rates ranging from 76 to 85% for several types of analysis which are comparable with success rates described in studies analyzing the same time period and region. The short amplicon approach proves its validity as well since we observe an inverse relationship between amplicon size and amplification efficiency, which reflects the authentic properties of ancient DNA molecules.

Our protocol has both historical and archaeological significance, as it will allow us to investigate periods of Mediterranean and Balkan population history that require further genetic research. From the pre-Roman times to Greek colonization of the region in the 8th c. BC to the Phoenician settlement and expansion of Rome until Byzantine rule, numerous events of population replacement, cultural exchange, and political development took place in this region. The genetic analysis of ancient populations of these regions would allow the examination of genetic continuity and mixed ancestry, favoring one sex and population structure despite significant historical changes.

With ancient DNA research, it becomes clear that there were numerous population replacements and migrations at the time of the Roman Empire and people from the eastern Mediterranean region started to migrate to other places. Steppe ancestry was introduced into the population for the first time during the Bronze and Iron Ages and southern Italian Iron Age individuals showed great genetic variability, corresponding to the heterogeneous Roman Empire. The genetic effect of Greek colonization on local populations has shown different results.

It seems that this type of research requires advanced technology, as the methods of high-throughput sequencing are very demanding in terms of equipment and laboratory space, making it impossible to study large museum collections or excavations in a consistent manner. Thus, our protocol provides a much more effective alternative, which is feasible for laboratories equipped with basic molecular biology equipment. Our protocol demonstrates the capacity to analyze about 80% of skeletal remains dated within this historical period and located in the territory between Italy and Albania based on published DNA studies and our actual tests results.

It is important to note that success rate depends on the taphonomy process and conditions in which skeletons are preserved rather than their age, dating within the above mentioned period [6,12,17,21]. Our protocol allows genetic analysis of populations from the Italian archaeological collections and examine pre-Roman populations including the Etruscans, Daunians, Italic tribes, and Greek colonial settlements in Magna Graecia and Phoenician colonies along the coast, Romans from republican and imperial periods and the Byzantine and early medieval populations. Moreover, the genetic analysis of Iron Age Illyrian populations and Greek colonial foundations in Albania such as Apollonia and Epidamnos and Roman provincial populations and subsequent Byzantine populations of Albanian and Balkan collections is possible.

It is essential that a standardized protocol combines all of these parameters – species and sex authentication and uniparental genetic markers and phenotypic traits, providing a great help for archaeological genetics due to decreasing methodological separation and provision of reliable primer sequences and research protocol applicable to all scientific community members. With this protocol, scientists of different disciplines can explore prehistory and the history of these regions combining genetic data with archaeological, historical, and anthropological, as well as bioarchaeological data, constructing the full picture of population movement, social organization, and life in the Mediterranean and Balkan territories throughout 2500 years.

With the current study, we developed the protocol applicable to Italian and Balkan archaeological collections as it would provide researchers with tools to solve some of the basic questions related to the effects of Greek colonization on demographic patterns and population replacement and maintenance due to the expansion of Rome, movement according to sex, genetic composition of pre-Roman indigenous groups, and demographic shifts due to cultural changes. This research gives rise to a comprehensive research framework that will facilitate international genetic research of Mediterranean and paleo-Balkan archaeological sites and make comparative studies in the Adriatic basin possible.

## Supplementary Material

Supplementary Material Details
