# Supplementary material for: Development and Validation of PCR-Based Ancient DNA Analysis for Italian and Western Balkan Archaeological Collections
Source: Balkan J Med Genet. 2026 Oct 14;29(1):39–50. doi: 10.2478/bjmg-2026-0008 (PMC13667942; doi:10.2478/bjmg-2026-0008)
Supplement: Supplementary file 1 [file bjmg-2026-0008-supplementary_materials.pdf]

BJMG 29 (1), 2026 • 1-4
DOI: 10.2478/bjmg-2026-0008

DEVELOPMENT AND VALIDATION OF PCR-BASED ANCIENT DNA ANALYSIS FOR ITALIAN AND WESTERN BALKAN ARCHAEOLOGICAL COLLECTIONS

Imran M[1], Capodicasa N[2], Barbacovi V[4], Maltese PE[3*], Bertelli M[1,3,5]

[*]**Corresponding Author:** Paolo Enrico Maltese, MAGI'S LAB, 38068 Rovereto, Italy,
Email: paolo.maltese@assomagi.org

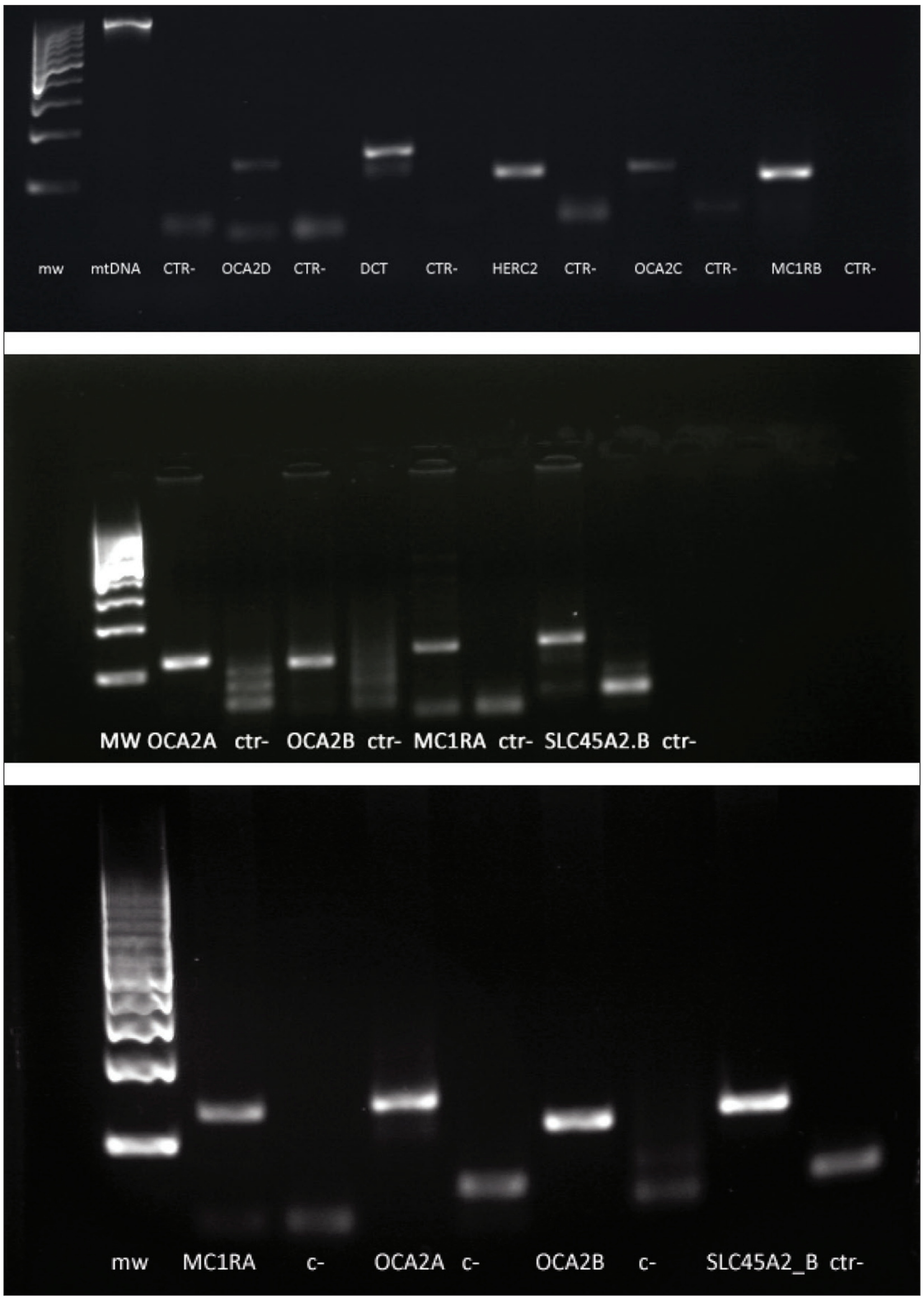

Figure S1. Agarose gel electrophoresis separate PCR products which contained pigmentation-related markers from ancient skeletal samples. The lanes of the test extend from the left side to the right side: molecular weight marker (MW); OCA2A sample with its negative control (ctr−); OCA2B sample with its negative control (ctr−); MC1RA sample with its negative control (ctr−); and SLC45A2_B sample with its negative control (ctr−).

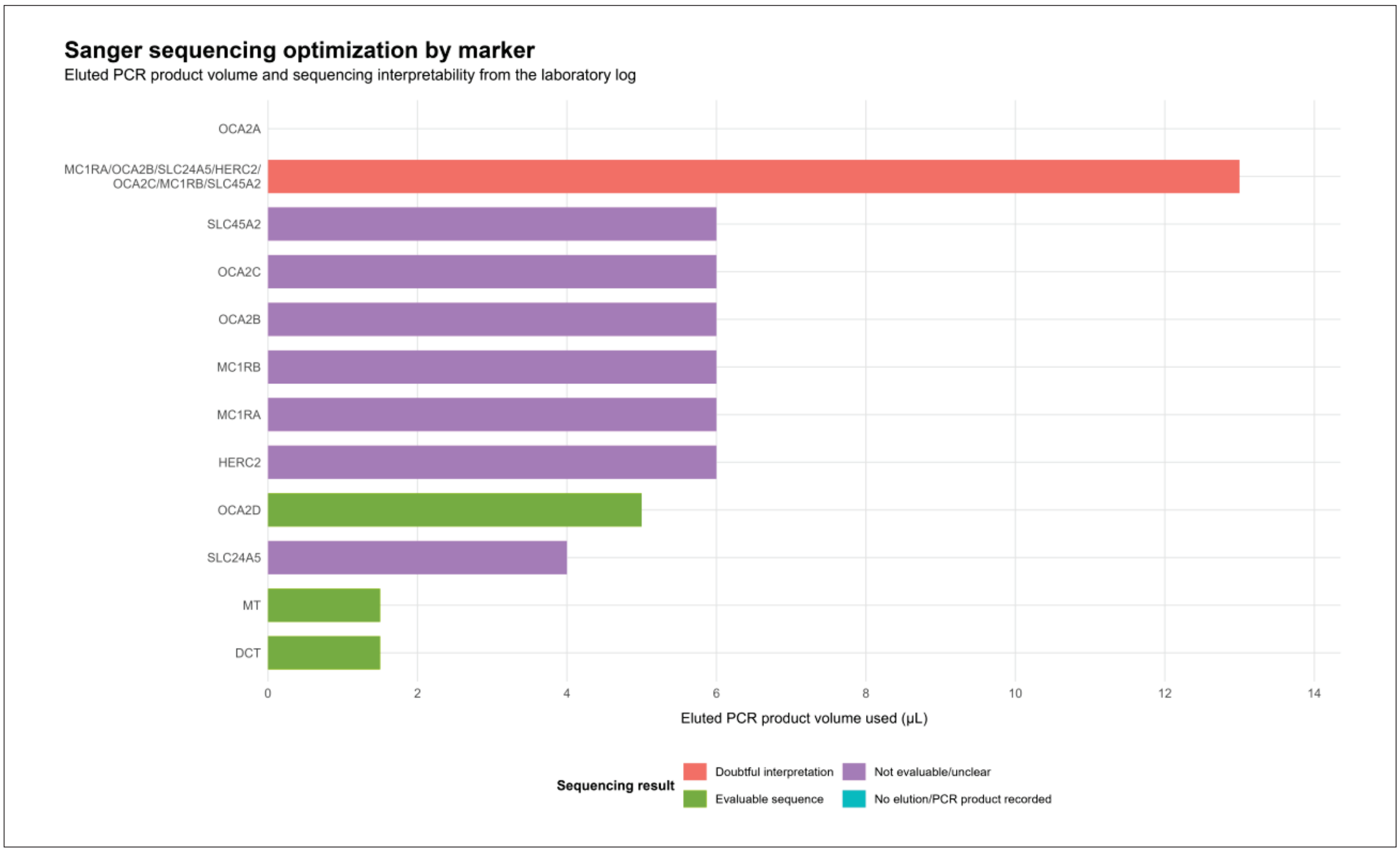

Figure S2. Optimization of Sanger sequencing by marker panels.
The horizontal bars represent the volume of eluted PCR product used for sequencing, while the colors represent how well the sequencing results could be interpreted. O*CA2A* was missing elution and PCR product; hence, there is no horizontal bar for that sample. *OCA2C* and *MT* were historical optimization assays not included in the final targeted panel.

Table S1. Targeted genes and their specified primers.

Analysis Step	Target	Primer Name	Primer Sequence (5'-3')	Amplicon Size (bp)	Information Provided
**Genetic Analysis**					
**1. Species Authentication**	16S rRNA	16S-HF	ATAACACGAGAAGACCCT	~100	Distinguishes human from animal remains; confirms sample is human origin
		16S-HR1	CCCACGGTCGCCCCAAC		
		16S-HR2	CCCGCGGTCGCCCCAAC		
**2. Sex Determination**	Amelogenin	Amelogenin-F	ACCTCATCCTGGGCACCCTGG	60	Identifies biological sex (XX=female, XY=male) via length polymorphism
		Amelogenin-R	AGGCTTGAGGCCAACCATCAG		
**3. Phenotypic Prediction - Hair Color**	MC1R rs1805007	MC1RA-F	CCGCTACATCTCCATCTTCTACGC	110	Red hair variant; contributes to hair color prediction
		MC1RA-R	GCGTGCTGAAGACGACACTGG		
	MC1R rs1805008	MC1RB-F	ATCTTCTACGCACTGCGCTACCAC	102	Red hair variant; contributes to hair color prediction
		MC1RB-R	GAAGAGCGTGCTGAAGACGACAC		
**4. Phenotypic Prediction - Eye Color**	HERC2 rs12913832	HERC2-F	TTGTTCTTCATGGCTCTCTGTGTCTG	108	Major determinant of blue vs. brown eye color
		HERC2-R	AGAGAAGCCTCGGCCCCTGA		
	OCA2 rs7495174	OCA2A-F	AGGCCCAGGCGGACTCAG	133	Eye color variation; works with HERC2
		OCA2A-R	AGGCAGGGAGGGTTTACACAGC		
	OCA2 rs6497268	OCA2B-F	CCAGGATTCAAAAAGAAAGTCTCAAGG	115	Eye color variation; additional OCA2 marker
		OCA2B-R	TTTCCTCCCATCACTGATTTAGCTG		
**5. Phenotypic Prediction - Skin Color**	SLC24A5 rs1426654	SLC24A5-F	CCCTTGGATTGTCTCAGGATG	118	Major determinant of light vs. dark skin pigmentation
		SLC24A5-R	TGAGTAAGCAAGAAGTATAAGGAGCAA		
	SLC45A2 rs16891982	SLC45A2-F	CCCTATAGTGCACACAACTCCACAGAG	96	Skin pigmentation variation
		SLC45A2-R	TGAGGAAAACACGGAGTTGATGC		
**6. Phenotypic Prediction - Hair/ Skin**	DCT rs2031526	DCT-F	CCTTGAATTGCTCTTGAAAAACTAA	149	Hair and skin pigmentation
		DCT-R	CAGCCCAATGATACACTTTCATTTAAC		
**7. Maternal Ancestry**	mtDNA HVR-I	HVR-I-F	CTCCACCATTAGCACCCAAAGC	120	Maternal haplogroup classification (H, U, J, T, K, V); traces maternal lineages and population origins
		HVR-I-R	GAGGATGGTGGTCAAGGGAC		
	mtDNA HVR-II	HVR-II-F	CACCATCCTCCGTGAAATCA	120	Maternal haplogroup classification; complements HVR-I data
		HVR-II-R	GGTGATGTGAGCCCGTCTAA		
**8. Paternal Ancestry (Males Only)**	Y-chr R1b-M269	R1b-M269-F	GAGTCTGTGCAGTTGATTCC	100	Bronze Age migrations; Western European ancestry
		R1b-M269-R	CTGAGCTGAGTTGGGTGTTC		
	Y-chr E1b1b-M78	E1b1b-M78-F	TCCAGGATTCTCTTCTTGCTG	100	North African/Mediterranean contacts; Neolithic expansion
		E1b1b-M78-R	CAGCTTGGCTAGGTAGTTGG		
	Y-chr J2-M410	J2-M410-F	ACTGTTGGAGGGTTGTAGAG	100	Near Eastern agricultural expansion; Greek/Phoenician colonization
		J2-M410-R	CCTCCTGCAGTGTTGTAGTT		

Table S2. Success rate based on analysis module and outcome.

analysis module	outcome	denominator group	success percent	success_percent_final	label
Overall amplification	Amplifiable DNA recovered	Ancient skeletal samples	100	100	100%
Sex determination	Complete sex profile	Ancient samples	85	85	85%
Species authentication	Human/non-human distinction	Tested samples	100	100	100%
Pigmentation profiling	Complete pigmentation SNP profile	Ancient samples	78	78	78%
Mitochondrial haplogroup	HVR-I/HVR-II haplogroup assigned	Ancient samples	82	82	82%
Y-chromosome haplogroup	Y haplogroup determined	Male ancient samples	76	76	76%
Reproducibility	Concordance across independent extractions/amplifications	Repeated assays	96	96	96%
Negative controls	Negative controls remained negative	All negative controls	100	100	100%

Table S3. Success rate based on fragment size groups.

fragment_size_group	description	success_percent	success_percent_final	label
<110 bp	Short amplicons	92	92	92%
>130 bp	Longer amplicons	68	68	68%

Table S4. Amplicon size by analysis modules and markers. Additional assays performed during Sanger sequencing optimization are presented in Figure S2 that were not included in the final targeted marker panel.

analysis_module	gene_or_target	marker_id	snp_or_region	amplicon_bp	information_provided
Species authentication	16S rRNA	16S-HF/16S-HR1/HR2	16S mitochondrial/rDNA vertebrate marker	100	Human vs non-human authentication
Sex determination	Amelogenin	Amelogenin-F/R	Amelogenin length polymorphism	60	Biological sex determination
Sex determination	SRY	SRY marker	Y-specific sex marker	60	Biological sex determination in males
Pigmentation	MC1R	MC1RA	rs1805007	110	Hair/red-hair variant
Pigmentation	MC1R	MC1RB	rs1805008	102	Hair/red-hair variant
Pigmentation	OCA2	OCA2A	rs7495174	133	Eye-color marker
Pigmentation	OCA2	OCA2B	rs6497268	115	Eye-color marker
Pigmentation	OCA2	OCA2D	OCA2 additional marker	144	Eye-color marker; evaluable sequence in notebook
Pigmentation	HERC2	HERC2	rs12913832	108	Major eye-color marker
Pigmentation	SLC24A5	SLC24A5	rs1426654	118	Skin pigmentation marker
Pigmentation	SLC45A2	SLC45A2	rs16891982	96	Skin pigmentation marker
Pigmentation	DCT	DCT	rs2031526	149	Hair/skin pigmentation marker; evaluable sequence in notebook
Maternal ancestry	mtDNA	HVR-I	mtDNA HVR-I	120	Maternal haplogroup classification
Maternal ancestry	mtDNA	HVR-II	mtDNA HVR-II	120	Maternal haplogroup classification
Paternal ancestry	Y-chromosome	R1b-M269	R1b-M269	100	Paternal haplogroup marker
Paternal ancestry	Y-chromosome	E1b1b-M78	E1b1b-M78	100	Paternal haplogroup marker
Paternal ancestry	Y-chromosome	J2-M410	J2-M410	100	Paternal haplogroup marker
